# Habituation of reinforcer effectiveness

**DOI:** 10.3389/fnint.2013.00107

**Published:** 2014-01-09

**Authors:** David R. Lloyd, Douglas J. Medina, Larry W. Hawk, Whitney D. Fosco, Jerry B. Richards

**Affiliations:** ^1^Research Institute on Addictions, State University of New York at BuffaloBuffalo, NY, USA; ^2^School of Medicine and Biomedical Sciences, State University of New York at BuffaloBuffalo, NY, USA; ^3^Department of Psychology, State University of New York at BuffaloBuffalo, NY, USA

**Keywords:** ADHD, behavioral regulation, dopamine, drug addiction, obesity, operant conditioning, psychomotor stimulant, sensory reinforcement

## Abstract

In this paper we propose an integrative model of habituation of reinforcer effectiveness (HRE) that links behavioral- and neural-based explanations of reinforcement. We argue that HRE is a fundamental property of reinforcing stimuli. Most reinforcement models implicitly suggest that the effectiveness of a reinforcer is stable across repeated presentations. In contrast, an HRE approach predicts decreased effectiveness due to repeated presentation. We argue that repeated presentation of reinforcing stimuli decreases their effectiveness and that these decreases are described by the behavioral characteristics of habituation (McSweeney and Murphy, 2009; Rankin etal., 2009). We describe a neural model that postulates a positive association between dopamine neurotransmission and HRE. We present evidence that stimulant drugs, which artificially increase dopamine neurotransmission, disrupt (slow) normally occurring HRE and also provide evidence that stimulant drugs have differential effects on operant responding maintained by reinforcers with rapid vs. slow HRE rates. We hypothesize that abnormal HRE due to genetic and/or environmental factors may underlie some behavioral disorders. For example, recent research indicates that slow-HRE is predictive of obesity. In contrast ADHD may reflect “accelerated-HRE.” Consideration of HRE is important for the development of effective reinforcement-based treatments. Finally, we point out that most of the reinforcing stimuli that regulate daily behavior are non-consumable environmental/social reinforcers which have rapid-HRE. The almost exclusive use of consumable reinforcers with slow-HRE in pre-clinical studies with animals may have caused the importance of HRE to be overlooked. Further study of reinforcing stimuli with rapid-HRE is needed in order to understand how habituation and reinforcement interact and regulate behavior.

## INTRODUCTION

The central theme of this paper is that an understanding of habituation of reinforcer effectiveness (HRE) provides important insight into the regulation of behavior by reinforcing consequences. This insight is valuable from a theoretical perspective because it provides a more accurate and parsimonious characterization of behavioral phenomena than current theories. Understanding HRE is also valuable because it can be used to improve the understanding and treatment of behavioral dysregulation including: attention-deficit/hyperactivity disorder (ADHD), autism spectrum disorder (ASD), obesity, and drug addiction.

Habituation theory and reinforcement theory make opposing predictions about the effects of reinforcing consequences on behavior. Simply stated, both frameworks describe how an organism responds to the experience of a stimulus. By definition in reinforcement, the repeated presentation of a reinforcing stimulus increases the behavior that produces it, and by definition in habituation, the repeated presentation of a stimulus decreases the behavior observed in response to the stimulus. Therefore, in reinforcement, repeated application of a stimulus is predicted to *increase* a behavior, but in habituation, repeated application of a stimulus is predicted to *decrease* a behavior. While research with animals has consistently indicated that increasing reinforcement frequency increases response rate, a more precise description of the actual relationship between reinforcing stimuli and behavior can be achieved through incorporation of concepts from habituation theory.

Any stimulus capable of serving as a reinforcer has an inherent associated sensory component which may be subject to habituation. Placing food in your mouth, flipping a switch, looking to your left or right, reaching out a hand toward another person, or moving a pen or highlighter across a piece of paper all produce changes in your sensory environment ….sights, sounds, smells, feelings, or tastes. Some of these experiences are novel, some have been previously experienced, some are subjectively good or bad, but all of these sensory components are intrinsically associated with experienced stimuli, and are able to affect the probability of reoccurrence of the response that produced them. It seems likely that these kinds of operant responses and weak reinforcing consequences are typical of the majority of reinforcing contingencies that regulate daily behavior. It has been assumed that the regulation of behavior by weak sensory events with primary reinforcing effects parallels the regulation of behavior by strong consumable reinforcers such as food and water in restricted animals. In this paper we suggest that rapid HRE differentiates weak sensory reinforcers from stronger consumable reinforcers.

Research using powerful consumable reinforcers such as food and water has uniformly reported a positive association between response and reinforcement rate. The positive relationship between response and reinforcement rate has been called the matching law (Herrnstein, 1974; de Villiers and Herrnstein, 1976; Heyman, 1983). In contrast, using a weak sensory reinforcer (light-onset), we have reported a negative association between response and reinforcement rate (Lloyd et al., 2012a). We believe that this opposite result was obtained because the effectiveness of light-onset habituates much more rapidly than the reinforcing effectiveness of consumable reinforcers such as food and water. Our interpretation of these results is that the reinforcing effectiveness of light-onset is overpowered by rapid HRE of light-onset.

Sensory reinforcement is understudied. Because most laboratory research with animals uses strong consumable reinforcers such as food and water, sensory reinforcers may be thought of as a “special case.” We do not agree. Although weak and transient, sensory reinforcers are important because they are pervasively integrated into our daily experiences. There are an almost infinite number of sensory and social stimuli that may act as sensory reinforcers. We believe that the majority of reinforcers that regulate daily activity are sensory reinforcers. Everything from smiles and praise to flipping a light switch to a car turn signal acts as a source of sensory reinforcement. It may be that the importance of HRE has not been recognized because of the almost exclusive use of powerful consumable reinforcers in pre-clinical studies of reinforcement processes; however, HRE of sensory reinforcers has important implications for both experimental research and applied clinical work with human populations.

### DEVELOPMENT OF THE HRE HYPOTHESIS

There is strong pre-clinical evidence that the effectiveness of reinforcers habituates. Over the past two decades, Frances McSweeney and her students have published a series of reports and reviews about HRE. In early reports, McSweeney (McSweeney, 1992; McSweeney and Hinson, 1992; McSweeney and Roll, 1993; McSweeney and Johnson, 1994; McSweeney et al., 1994a,b,1995a,b,c,d,e,1996e; Cannon and McSweeney, 1995; Roll et al., 1995; Weatherly and McSweeney, 1995; Weatherly et al., 1995) observed within-session decreases in operant responding which she later hypothesized may be due to HRE (McSweeney et al., 1996a,b,c,d; Weatherly et al., 1996). Previous to McSweeney’s development of the HRE hypothesis, these within-session decreases in responding were commonly explained as being the result of physiological satiation. The research program conducted by McSweeney and her students has provided convincing evidence that within-session changes in operant responding are influenced by habituation and that explanations based on physiological satiation are unlikely. The HRE hypothesis developed by McSweeney and her students has the potential to have a major impact on our understanding of basic reinforcement mechanisms and the application of reinforcement to clinical problems. Objectives of this paper are: (1) to push the HRE perspective beyond consumable reinforcers (e.g., food or water) with consideration of a broader range of sensory reinforcers, (2) to examine the effects of stimulant drugs on HRE, (3) to develop an initial neural/behavioral model of HRE, and (4) to consider the possible clinical impact of HRE.

### TEN BEHAVIORAL CHARACTERISTICS OF HABITUATION

The current understanding of behavioral habituation is well-described by a list of 10 empirical characteristics identified in previous research (McSweeney and Murphy, 2009; Rankin et al., 2009). This list of characteristics is predominately derived from behavioral models where the habituating stimulus occurs *before* a behavior and causes (elicits) the behavior. This differs from operant conditioning where the habituating stimulus occurs *after* a behavior is emitted and is a direct consequence of that behavior. In both cases repeated stimulus presentation leads to a decreased behavioral response, and there is no *a priori* reason to think the habituation characteristics do not describe both behavioral models.

The 10 characteristics are listed in **Table 1** along with a prediction that each characteristic makes when used to describe HRE. All of these predictions are empirically testable, and in some cases are opposite of the prediction of current reinforcement theory. For example, characteristic #4 in **Table 1** predicts that more frequent presentation of a reinforcer will decrease response rate. This contradicts the widely accepted prediction of the matching law (Herrnstein, 1974; de Villiers and Herrnstein, 1976; Heyman, 1983) in which rate of responding is positively related to the rate of reinforcement. As was previously mentioned, we have reported that the rate of responding for a sensory reinforcer (light-onset) can be negatively associated with reinforcement rate (Lloyd et al., 2012a; see discussion in characteristics section).

**Table 1:** Predictions for habituation of reinforcer effectiveness made by the 10 behavioral characteristics of habituation^1^ as described by Rankin et al. (2009).

#	Habituation characteristic	Habituation of reinforcing effectiveness
1	Repeated application of a stimulus results in a progressive decrease in some parameter of a response to an asymptotic level.	Predicts that repeated presentation of a reinforcer will cause a within-session decline in response rate.
2	If the stimulus is withheld after response decrement, the response recovers at least partially over the observation time (“spontaneous recovery”).	Predicts that a subject responding for a reinforcer in 2 consecutive testing sessions with a long break between sessions will show greater responding during the start of the second session than at the end of the first.
3	After multiple series of stimulus repetitions and spontaneous recoveries, the response decrement becomes successively more rapid and/or more pronounced (this phenomenon can be called potentiation of habituation).	Predicts that a subject responding for a reinforcer in once per day sessions for five consecutive days will show a faster within-session decline in response rate on the 5^th^ day than on the 1^st^ day of testing.
4	More frequent stimulation results in more rapid and/or more pronounced response decrement and more rapid spontaneous recovery.	Predicts that a subject responding for a reinforcer according to a Fixed Interval (FI) 10 s schedule will show a greater within-session decrease in responding than a subject responding for a reinforcer on a FI 100 s schedule.
5	Within a stimulus modality, the less intense the stimulus, the more rapid and/or more pronounced the behavioral response decrement. Very intense stimuli may yield no significant observable response decrement.	Predicts that a subject responding for a large magnitude reinforcer will show less within-session decline in responding than a subject responding for a smaller magnitude reinforcer.
6	The effects of repeated stimulation may continue to accumulate even after the response has reached an asymptotic level. This effect of stimulation beyond asymptotic levels can alter subsequent behavior, for example, by delaying the onset of spontaneous recovery.	Predicts that a subject that responds for a reinforcer until an asymptotic baseline (operant) level of responding is reached will show greater initial responding upon retest than a subject that is left in the test situation for additional testing after asymptotic responding is reached.
7	Within the same stimulus modality, the response decrement shows some stimulus specificity.	Predicts that changing the stimulus properties of the reinforcer after responding has declined (habituated) will increase responding.
8	Presentation of a different stimulus results in an increase of the decremented response to the original stimulus. This phenomenon is termed “dishabituation.”	Predicts that after responding for a reinforcer has declined (habituated), the introduction of a separate non-contingent novel stimulus will increase responding for the reinforcer.
9	Upon repeated application of the dishabituating stimulus, the amount of dishabituation produced decreases.	Predicts that repeated dishabituation by a non-contingent stimulus (see prediction #8) will have diminished effects on responding with each successive use.
10	Some stimulus repetition protocols may result in properties of the response decrement that last hours, days, or weeks. This persistence of aspects of habituation is termed long-term habituation.	Predicts that, with repeated testing, total responding during daily test sessions will decrease and that this decrease in responding will be long lasting.

1 The descriptions of the characteristics of habituation described in this table are abbreviated in order to save space. If clarification is needed please refer to the original descriptions provided by Rankin etal. (2009).

### SENSORY AND BIOLOGICALLY IMPORTANT REINFORCERS

We initially became interested in HRE while conducting research on sensory reinforcement. We found that responding for sensory reinforcers such as light-onset showed marked within-session declines (Gancarz et al., 2012a; Lloyd et al., 2012a). If we had observed these decreases in responding using a reinforcing stimulus which was consumed (e.g., food or water), we may have attributed the decreases to physiological satiation (i.e., the animals were less hungry or thirsty). However, since light-onset is not consumed, decreases in its reinforcing effectiveness cannot be explained by physiological satiation. In the absence of a satiation mechanism, we turned to McSweeney’s HRE hypothesis to explain the observed decreases. As is described later in section “Experimental Analysis of HRE with a Light Reinforcer,” we have found a strong correspondence between several predictions listed in **Table 1** and operant responding for light-onset.

Glanzer (1953) was the first to attempt a precise definition of sensory reinforcement-related phenomena. His idea was that exposure to a particular sensory stimulus led to the development of a “quantity of stimulus satiation” specific to that stimulus. With repeated experience of the stimulus, this “quantity” increased and the tendency to respond to the associated stimulus decreased. In their landmark review of habituation, Thompson and Spencer (1966) point out that Glanzer’s stimulus satiation hypothesis can be, “viewed as a formalized restatement of some of the parametric characteristics of habituation.” The strong similarity of the characteristics of Glanzer’s stimulus satiation hypothesis to the characteristics of habituation described by Thompson and Spencer indicates that habituation has been relevant to even the earliest considerations of sensory reinforcement-related phenomena.

Many researchers have followed Glanzer’s example and have referred to what may have been habituation related decreases in responding as satiation. For example decreases in responding for social reinforcers have sometimes been attributed to satiation of a need for social reinforcement. A review by Eisenberger (1970) of studies exploring the extent to which task performance was enhanced by the provision of social praise indicated that individuals who were praised by the experimenter before the start of the task were less responsive to social reinforcers (i.e., had less improvement in performance) than participants who received no praise before the task. An HRE interpretation suggests that the performance decrement is due to a greater number of reinforcer presentations and not satiation of a need for social reinforcement. Unfortunately, the study designs do not allow for clear interpretation of whether satiation or habituation can better account for the data, but this example highlights the relevance of habituation for reinforcers that are highly salient for humans.

Sensory reinforcers are most often defined as reinforcers that are not biologically important. For example, Kish (1966) defined sensory reinforcement as a “primary reinforcement process resulting from the response-contingent presentation or removal of stimuli of moderate intensity which are not related to some organic need, or removal of aversive stimulation.” In another review, Eisenberger (1972) defined sensory reinforcers as “incentives that have no evident tissue-maintenance or reproductive functions.” In recent reports, we have referred to sensory reinforcers as “sensory events that do not reduce tissue needs” (Gancarz et al., 2011), as “reinforcers that do not affect homeostatic balance” (Gancarz et al., 2012a), and as “reinforcers that are not biologically important” (Lloyd et al., 2012a). The problem with these types of definitions is that “biological importance or significance” can only be defined very generally and is hard to precisely measure.

With this in mind, we suggest that reinforcers lie along a continuum with the most significant biological reinforcers such as food, water, and painful stimuli at one end of the spectrum and those sensory stimuli, which are often described as “neutral” or “indifferent^1^,” at the opposite end of the spectrum. Stimuli at both ends of the spectrum of biological significance can evoke reflexive responses. For example, food in the mouth elicits salivation, and onset of a light elicits orienting responses. The topographies of these reflexive reactions are markedly different, but perhaps an even more important difference is that orienting responses to the light habituate more rapidly than salivation to food in the mouth of a food restricted animal. When considered as a reinforcing consequence, we expect that the reinforcing effectiveness of light-onset would habituate more rapidly than that of food. We hypothesize that the rate of HRE may distinguish between sensory and biologically important reinforcers and that HRE occurs across the entire continuum.

For consumable reinforcers such as food and water, HRE and physiological satiation provide competing explanations for within-session declines in responding. A water deprived rat may show a within-session decrement in responding because the water previously consumed during the test session alters intra- and/or extracellular fluid levels. It is difficult to completely disregard satiation-based explanations. However, it is possible to support the alternative HRE explanation by using stimulus specificity (characteristic 7) and dishabituation (characteristic 8) tests to rule out satiation-based accounts. For a thorough discussion of various approaches to disentangling satiation- and habituation-based accounts of within-session declines in responding, see McSweeney and Murphy (2009) and Epstein et al. (2009).

In agreement with McSweeney and Murphy (2009), our view is that water and food in the mouth are sensory stimuli that have varying reinforcing effects. Restricting access to water and food is an establishing (motivating) operation that controls the reinforcing effectiveness of the sensory stimuli that are associated with food and water in the mouth (Murphy et al., 2003). In addition to food or water restriction, post-absorptive effects of ingested stimuli may affect the reinforcing effectiveness of predictive sensory stimuli (de Araujo et al., 2012). However, regardless of the level of satiation, or the association of sensory stimuli with post-absorptive factors, it is the contention of this review that the reinforcing effects of sensory stimuli habituate. In the case of sensory reinforcers such as light-onset, satiation or post-absorptive effects do not provide a competing explanation for within-session declines in responding. According to the continuum hypothesis, the effectiveness of all reinforcers (indifferent or biologically important) is strongly regulated by their immediate sensory effects.

### STIMULANT DRUGS AND DOPAMINE

Our interest in sensory reinforcement emerged following reports that sensory reinforcers such as light-onset play an important role in the reinforcing effects of the stimulant drug nicotine (Donny et al., 2003a). As is described in the stimulant section below, psychomotor stimulants increase responding for sensory reinforcers, and sensory reinforcers may play an important role in the reinforcing effects of stimulant drugs. We believe that stimulant drug-induced changes in the reinforcing effectiveness of sensory reinforcers are regulated by dopamine (DA). This hypothesis is supported by Redgrave and Gurney (2006) who described a DA-based neural system that controls the reinforcing effects of sensory reinforcers. This theory is described in more detail in section “Neural/Behavioral Model of HRE.”****

Based on our work with stimulant drugs and sensory reinforcement, we have hypothesized that stimulant drugs disrupt (slow) expression of HRE and that disruption of HRE is an important determinant of stimulant drug effects on operant responding (Lloyd et al., in press). Additionally, the effects of stimulant drugs may depend on the rate of reinforcer habituation, with stimulants having a greater effect on rapidly habituating reinforcers (i.e., sensory-reinforcers such as light-onset) as compared to slowly habituating reinforcers (i.e., biologically important reinforcers such as food and water).

### CLINICAL IMPLICATIONS OF HRE

To date there has been little intentional application of the HRE concept in the clinic. However, many clinical treatment protocols reflect the unintentional use of HRE. An example of unintentional application of HRE in clinical treatment is the common use of reinforcer menus, which allow reinforcing consequences to be varied. From experience, clinicians have learned that varying reinforcers (which prevents HRE) results in greater overall maintenance of the desired behavior (i.e., reinforcer effectiveness). This example of unintentional use of the HRE concept and others are discussed in section “Clinical Significance of HRE.”

An example of intentional application of HRE concepts to solve a clinical problem is the innovative research of Leonard Epstein and co-workers who have hypothesized that slow HRE may play an important role in obesity. Epstein’s group has repeatedly demonstrated that patterns of within-session responding for food are controlled by stimulus specificity (**Table 1**, characteristic #7). In these demonstrations, responding for one type of food is observed to decrease with repeated food presentation, but can be increased by introduction of a new food (Wisniewski et al., 1992; Myers Ernst and Epstein, 2002; Epstein et al., 2003, 2008, 2010; Temple et al., 2007a,b, 2008b). This pattern of intake has also been referred to as sensory-specific satiation, which Epstein et al. (2009) argued is a special case of habituation. These authors note that habituation theory predicts that non-food environmental factors, which are not considered by the sensory-specific satiety concept, influence food intake. For example, Temple et al. (2007a) reported that watching TV while eating causes dishabituation and increases food intake. Evidence that habituation is a determinant of food intake, and that habituation (not satiation) is responsible for within-session declines in responding is extremely important because it implicates a variety of non-food-related environmental factors in the problem of obesity. These environmental factors are frequently not considered by current approaches. In further support of the hypothesis that slow-HRE contributes to obesity Epstein’s group has reported that overweight children exhibit decreased habituation to food (Temple et al., 2007b; Epstein et al., 2008), and that future increases in body mass index can be predicted by low food habituation rates (HRs; Epstein et al., 2011).

Clinical examples of dysfunctional behavioral regulation (i.e., obesity, ADHD, ASD, and drug addiction) are discussed throughout the manuscript and in more depth in section “Clinical Significance.”

## EXPERIMENTAL ANALYSIS OF HRE WITH A LIGHT REINFORCER

Following reports that nicotine enhances the reinforcing effectiveness of light-onset (Donny et al., 2003a), we began studying the interaction of the sensory reinforcing effects of a visual stimulus (VS) with the reinforcing effectiveness of other stimulant drugs such as methamphetamine. We used snout-poking as an operant response because rats snout poke at a low rate without any training, allowing us to avoid the need for training the operant response with additional reinforcers such as food or water (cf., bar-pressing).

In our initial (unpublished) attempts to measure light-reinforced behavior, we employed methods that we had previously used to study operant responding for biologically important reinforcers. We placed rats in lighted test chambers and made 5 s of light-*offset* contingent upon snout-poking, according to a fixed ratio (FR1) schedule of reinforcement, with hour-long test sessions conduced 7 days a week to ensure animals had enough time to learn the contingency between responding and light-offset. The results of these initial studies were disappointing. The rate of active snout-poking failed to increase across days, and in fact decreased, and preference for the active snout-poke hole over the inactive hole was inconsistent.

Since this initial attempt to measure light reinforced behavior, we have learned a great deal about light-reinforced responding (Gancarz et al., 2011, 2012a,b; Lloyd et al., 2012a,b). In retrospect, we are able to appreciate some errors of approach we made during our first efforts. We have come to believe that sensory reinforcers are much weaker than consumable reinforcers, in part because the reinforcing effectiveness habituates much more rapidly for light than for consumable biologically important reinforcers. Our understanding of light reinforcement was aided by our gradual rediscovery of the substantial literature on light-reinforced behavior (for reviews see: Lockard, 1963; Kish, 1966; Berlyne, 1969; Tapp, 1969; Eisenberger, 1972). Here we highlight three key aspects of that work.

First, animals should be habituated to the test chamber prior to making light change contingent upon a response. Habituation to the test chamber enhances the effectiveness of light reinforcement (Appel and Hurwitz, 1959; Crowder, 1961; Leaton et al., 1963). Pre-exposure to the test chamber allows the reinforcing effectiveness of competing novel stimuli to habituate; so that when the response-contingent light stimulus eventually occurs it will be novel and/or surprising (and unhabituated) relative to other stimuli in the test chamber.

Second, shorter test sessions should be used in order to observe robust light reinforcement effect. Consistent with an HRE explanation, previous studies consistently show large within-session decrements in responding, with the most responding for the light stimulus occurring during the first minutes of the test session (Roberts et al., 1958; Premack and Collier, 1962; McCall, 1966; Tapp and Simpson, 1966; Gancarz et al., 2011; Lloyd et al., 2012a). Additionally, during hour-long test sessions, habituation may be so extensive that it prevents the spontaneous recovery (**Table 1**, characteristic #2) of responding which otherwise may have been observed in subsequent sessions (**Table 1**, characteristic #6).

Third, and relatedly, longer inter-session intervals should be employed. Increasing the between-session interval attenuates or prevents the decreases in response rate that occur with repeated testing (Forgays and Levin, 1961; Fox, 1962; Premack and Collier, 1962; Eisenberger, 1972). From an HRE perspective, this is spontaneous recovery (**Table 1**, characteristic #6).

The procedures that we initially used to measure the reinforcing effectiveness of light-onset probably would have detected robust effects of consumable reinforcers such as food or water. We believe that this difference is caused by relatively rapid-HRE of sensory reinforcers relative to that of slow-HRE consumable reinforcers like food and water (particularly in food- and water-restricted animals).

Some of the characteristics of habituation are counterintuitive when applied to reinforcement. A fundamental characteristic of habituation is that more frequent stimulation causes more rapid and/or pronounced decrements in responding. If we view the reinforcing effectiveness of light-onset as being a function of habituation, then this indicates that more frequent presentations of the reinforcer will result in a decreased rate of responding. In contrast, a large amount of empirical data from operant experiments using slow-HRE consumable reinforcers like food and water in food and water-restricted animals generally indicates that response rate increases as a function of reinforcer frequency (Herrnstein, 1974; de Villiers and Herrnstein, 1976; Heyman, 1983). This leads to two contradictory predictions regarding the effect of sensory reinforcer presentation rate on responding. An HRE-based hypothesis predicts that less frequent presentation of a reinforcer will decreases HRE and thereby increase response rate. In contrast, previous research with biologically important reinforcers such as food and water indicates that less frequent presentation of a reinforcer should decrease response rate.

**FIGURE 1:**
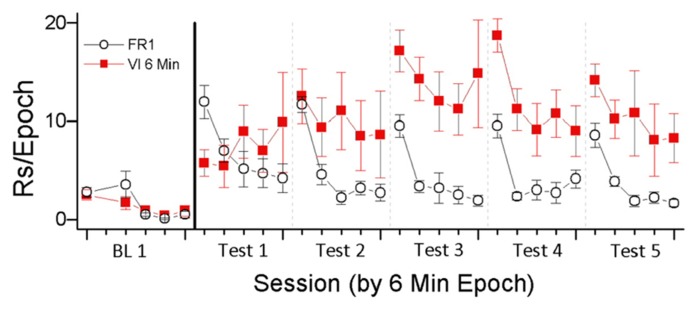
**In this experiment, the sensory reinforcer of light-onset was contingent on snout-poking.** Light was presented according to one of three schedules (FR 1, VI 1 min, and VI 6 min; data for VI 1 min not shown). The experiment had two phases, one in which animals were pre-exposed to an experimental chamber for 10 sessions, followed by the second phase of 10 sessions in which animals could snout-poke into one of two holes to produce 5 s light-onset. The data are depicted as two session blocks and show the rate of responding in 6 min epochs of each 30 min test session. BL 1 indicates responding at the end of the pre-exposure phase when there was no response-contingent light-onset. Tests 1–5 indicate two session blocks of light-contingent responding.

To investigate this relationship, we examined responding for light-onset presented according to FR1 and VI 6 min schedules of reinforcement (Lloyd et al., 2012a). On the FR 1 schedule, every snout-poke produced the VS (5 s light-onset). On the VI 6 min schedule, snout-poking produced light-onset on the average of every 6 min. The results are depicted in **Figure 1**. As predicted by the HRE hypothesis, higher rates of responding occurred in the VI 6 min condition, the condition in which reinforcement was *less* frequent. This result indicates the importance of HRE as a regulator of reinforcement and that the functional relationship between reinforcer rate and response rate may be different for non-consumable purely sensory reinforcers and consumable biologically important reinforcers. We believe that this difference is because sensory reinforcers habituate more rapidly than consumable reinforcers in deprived animals.

The data depicted in **Figure 1** illustrate a number of important characteristics of habituation described by Rankin et al. (2009) and listed in **Table 1**. The first behavioral characteristic of habituation (**Table 1**) is that, “repeated application of a stimulus results in a progressive decrease in some parameter of a response to an asymptotic level.” For the two reinforcement schedules shown in **Figure 1**, responding was greatest during the first 6 min epoch of the test session and then decreased. The FR1 schedule produced reliable within-session decreases in responding for all test sessions. The VI 6 min schedule produced reliable within-session decreases in responding during tests 3–5. For the FR1 schedule, where there are more repetitions of the reinforcer, the pattern of results is consistent with responding being reduced to “asymptotic levels.” In addition to the present data, previous studies have consistently shown both between- and within-session decrements in VS reinforced responding (Roberts et al., 1958; Premack and Collier, 1962; McCall, 1966; Tapp and Simpson, 1966; Gancarz et al., 2012a).

The second behavioral characteristic of habituation (**Table 1**) is that, “If the stimulus is withheld after response decrement, the response recovers at least partially (spontaneous recovery).” Following the increases in responding due to the initial primary reinforcing effects of the VS, there was clear evidence of spontaneous recovery from the decrements in responding that occurred during the previous test session. That is, responding during the first 6 min epoch of the test session is greater than responding during the last 6 min epoch of the previous test session. In addition, previous studies of the reinforcing effectiveness of visual stimuli have shown that increasing the intersession intervals results in greater recovery of responding (Forgays and Levin, 1961; Fox, 1962; Premack and Collier, 1962; Eisenberger, 1972). These data are consistent with the interpretation that longer intervals between test sessions result in greater spontaneous recovery of the reinforcing effectiveness of visual stimuli.

The third behavioral characteristic of habituation (**Table 1**) is that, “After multiple series of stimulus repetitions and spontaneous recoveries, the response decrement becomes successively more rapid and/or more pronounced.” For the FR1 schedule, between-session decreases in total session responding were observed for all test sessions following test session 1. For the VI 6 min schedule, between-session decreases in total session responding were observed for the four test sessions following test sessions 5 and 6. For both schedules, the within-session pattern of responding generally indicates that the decrease in responding from the first 6 min epoch to the second 6 min epoch became larger with repeated testing. Taken together, the data are consistent with the interpretation that within-session decreases in reinforcer effectiveness due to habituation are accelerated by repeated cycles of testing and recovery.

The fourth behavioral characteristic of habituation (**Table 1**) is that, “More frequent stimulation results in more rapid and/or more pronounced response decrements.” Consistent with this characteristic, the schedule providing the most frequent reinforcement (FR1) caused faster and more pronounced decreases in within-session responding than were observed in the schedule providing the least frequent reinforcement (VI6). This inverse relationship is consistent with the interpretation that the initial reinforcing effectiveness of the VS was decreased by the frequency of its occurrence. One interpretation of these data is that the initial primary reinforcing effectiveness of the VS was equivalent and that reinforcer effectiveness was decreased by habituation. The degree of habituation was determined by the schedule of reinforcement, with schedules that permitted higher frequencies of response-contingent VS presentation resulting in more rapid habituation.

The seventh behavioral characteristic of habituation (**Table 1**) is that, “Within the same stimulus modality, the response decrement shows *stimulus specificity.*” For HRE, the test for stimulus specificity is to present a stimulus contingent upon the response that has stimulus properties that are different from the original reinforcing stimulus. If the observed response decrements are caused by habituation and not by adaptation or fatigue, the animal should show increased responding to produce the novel stimulus.

**FIGURE 2:**
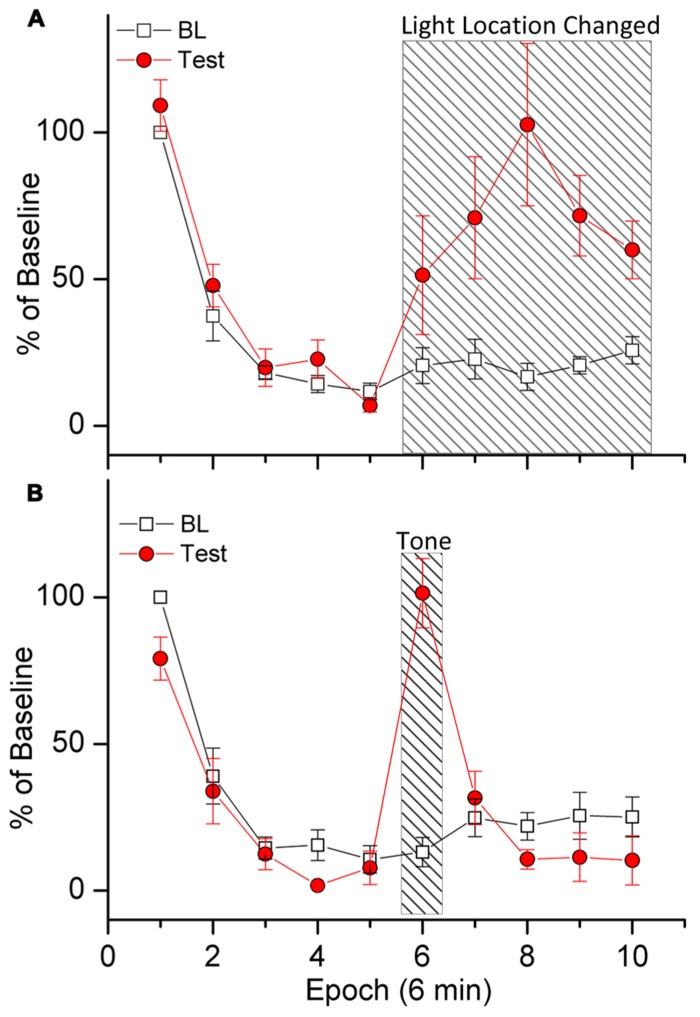
**Demonstration of stimulus specificity (A) and dishabituation **(B)**.** Rats were trained to respond for response-contingent light-onset presented in the front of the test chamber according to a VI 1 min schedule of reinforcement. Stimulus specificity **(A)** was tested by shifting the location of the response-contingent light to the rear of the test chamber during the last 30 min of the test session [indicated by slanted lines in **(A)**]. Dishabituation **(B)** was tested by turning on a loud warbling tone during minutes 30–36 of the test session [indicated by slanted lines in **(B)**]. The data are plotted in 10 6-min epochs for the 60 min test sessions. Baseline (BL) responding during each epoch is plotted as a percent of total responding during the first epoch. Test-day responding was divided by the number of responses during BL for each epoch.

Demonstrations of stimulus specificity are important because they provide a litmus test for determining if declines in responding can be attributed to habituation. Evidence for stimulus specificity of response decrements in light reinforced responding is presented in **Figure 2A** (Wang et al., in preparation). In this experiment we first trained rats to respond to produce light-onset that originated from a light located at the front of the test chamber. During a subsequent 1 h challenge test, subjects were able to respond to produce light in the front of the chamber for the first 30 min of the test session, and for the following 30 min of the test session, responding activated a light at the rear (instead of the front) of the test chamber. Robust within-session decreases in responding were observed, indicating HRE. Shifting the light to the rear of the chamber increased responding above BL levels during the last 30 min of the test session, demonstrating that the within-session decrease in responding was dependent upon specific stimulus properties of the light. As stated above, this is important because it shows that within-session decreases in responding were not caused by factors such as motor fatigue or sensory adaptation. Had these factors been responsible for the within-session decrease in responding, the change in location of the light would not have resulted in any increases.

The eighth behavioral characteristic of habituation (**Table 1**) is that, “Presentation of a different stimulus results in an increase of the decremented response to the original stimulus.” A test involving restoration of responding by introduction of a non-contingent external stimulus is termed a test of *dishabituation*. Evidence for dishabituation by a non-contingent external stimulus on responding for a sensory reinforcer is presented in **Figure 2B** (Wang et al., in preparation). A loud warbling tone was presented for 6 min during the middle of a 1 h test session (minutes 30–36). (Note that this is different from the stimulus specificity test above because the reinforcer was not changed). **Figure 2B** shows that responding was increased during presentation of the warbling tone. The data in **Figure 2B** are plotted in 10 6-min epochs for the 1 h test sessions. Robust within-session decreases in responses were observed, indicating HRE. Responding during each epoch is plotted as a percent of total responding during the first epoch. Baseline (BL) indicates responding during the two previous sessions. Test-session responding was divided by the number of responses during BL for each epoch. Presentation of the tone increased responding above BL levels during min 30–36 (epoch 6), ruling out motor fatigue or sensory adaptation.

Rankin et al. (2009) placed particular emphasis on stimulus specificity and dishabituation as evidence for habituation because both tests effectively rule out fatigue or sensory adaptation as explanations for decrements in responding. Previous studies have shown that both tests of stimulus specificity (Aoyama and McSweeney, 2001; Epstein et al., 2003; Kenzer et al., 2013) and tests of dishabituation (McSweeney and Roll, 1998; Aoyama and McSweeney, 2001; Kenzer et al., 2013) are able to restore responding for consumable reinforcers (see Epstein et al., 2009, for a review). The work reviewed demonstrates both phenomena also occur with sensory reinforcers in rodents.

Evidence that the reinforcing effectiveness of sensory (non-consumable) reinforcers also habituates in humans has been documented in a recent paper by Kenzer et al. (2013). This study used tests of stimulus specificity and dishabituation to demonstrate that within-session decreases in response rate were due to HRE for both food and two types of sensory reinforcers. In this study, participants clicked a square on a computer screen with a mouse. After each click the cursor controlled by the mouse was reset to the bottom corner of the screen. Click responses in the square were reinforced according to FR schedules of reinforcement. Different kinds of reinforcers were tested, including food items, social statements (e.g., “good job”) and pictures. When within-session response rate decreased to less than a third of the initial response rate, participants were exposed to a novel stimulus condition consisting of a change in one of the following: reinforcer value, reinforcer type, reinforcer amount, reinforcement schedule, or color on the computer screen, with no change used as a control. Eighty-nine percent of the participants who experienced a novel stimulus condition increased in responding relative to the immediate pre-novel stimulus response rate. These results are consistent with stimulus specificity and/or dishabituation, indicating that the observed declines in responding reflect habituation.

In this section we have provided evidence that sensory reinforcement is regulated by habituation related processes. We have described data indicating that decreases in responding for visual reinforcers in rodents are well-described by six of the 10 characteristics of habituation listed by Rankin et al. (2009). We have also provided evidence that HRE occurs for sensory reinforcers in human participants as well. McSweeney and coworkers (McSweeney et al., 1996a; McSweeney and Murphy, 2000, 2009) have provided similar evidence indicating that within-session changes in responding by animals responding for food and water reinforcers can also be explained by the characteristics of habituation. Finally, as was described in the previous section, Epstein and coworkers (Wisniewski et al., 1992; Myers Ernst and Epstein, 2002; Epstein et al., 2003, 2008, 2010; Temple et al., 2007a,b, 2008b) have repeatedly provided evidence supporting HRE for food reinforcers in humans.

## THE QUANTITATIVE MEASURE OF HRE

In order to more precisely characterize HRE, we have developed a method to quantify HR. Habituation assumes a declining rate of responding as a function of repeated stimulation. Our HR metric estimates the rate at which responding declines during a test session. Importantly, the HR measure is calculated so that absolute differences in response rate do not affect the HR estimate. As others (McSweeney et al., 1996a; Leussis and Bolivar, 2006) have pointed out, if differences in baseline responding are not taken into consideration, differences attributed to habituation may actually be due to baseline differences in absolute response levels.

**FIGURE 3:**
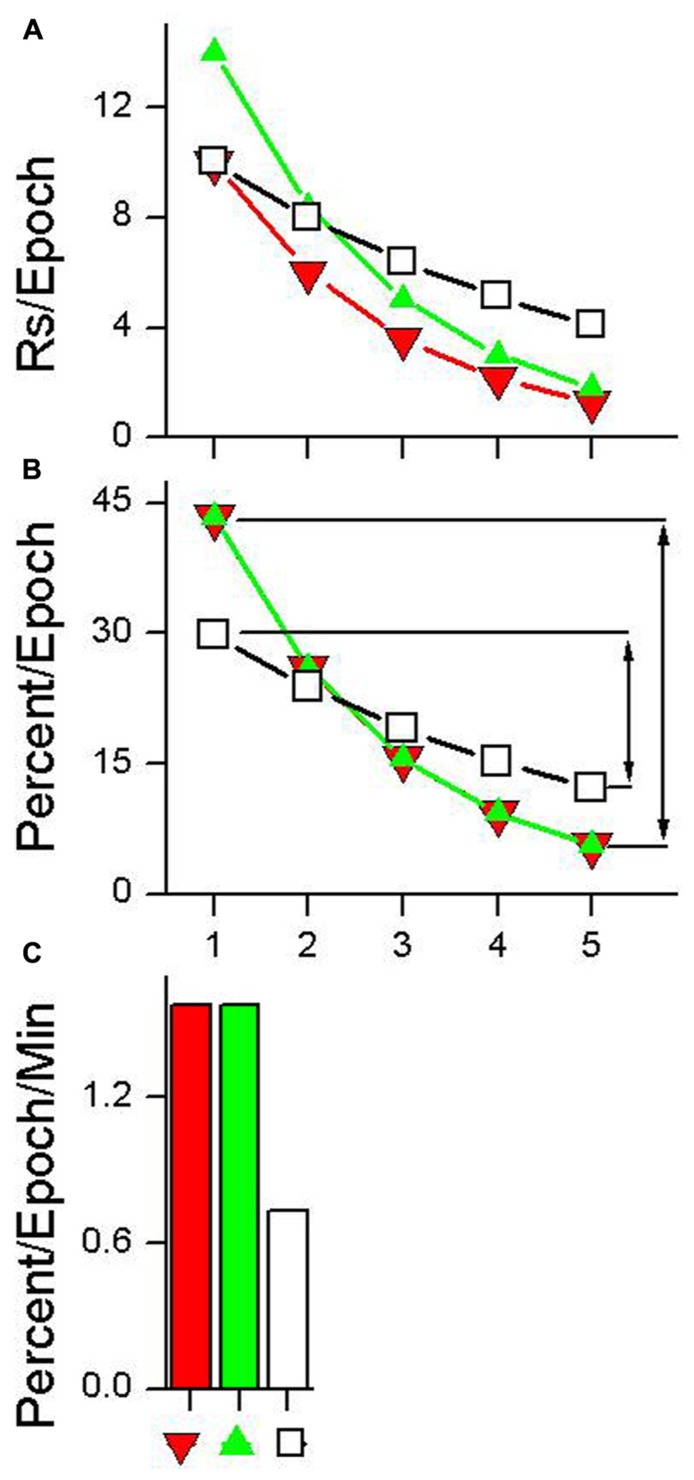
**Calculation of habituation rate (HR).** **(A)** Data having different absolute rates of responding plotted as five equal duration epochs. **(B)** The same data with each epoch plotted as a percentage of total responding. The lines with arrows in plot **(B)** indicate the difference between the first epoch of the test session and the epoch with the lowest percentage of responding. This difference is divided by the time between the first epoch and the epoch with the lowest percentage of responding to produce the HR measure shown in plot **(C)**. See text for details.

**Figure 3A** shows data from a hypothetical test session plotted using five epochs (choice of epoch length is arbitrary). Three different hypothetical examples of habituation are shown in **Figure 3A**. HR estimates the rate at which responding declines during the test session. Calculation of this metric is a three step process.

(1) Organize the data into epochs indicating the absolute rate of responding that occurred in each epoch, as is shown in **Figure 3A**.
(2) Convert the absolute responding per epoch measures shown in **Figure 3A** to a percentage of total-session responding as shown in **Figure 3B**. This can be done using the following equation:

$$\frac{Percent}{Epoch}=100\times \frac{Rs_{Epoch}}{TotalRs},$$

where Rs_Epoch_ is the total number of responses emitted during a given epoch and TotalRs is the total number of responses that occurred during the session. This transformation normalizes the data from habituation curves that have different absolute numbers of responses and produces habituation curves with equal areas under the curve. For example, the three habituation curves shown in **Figure 3A** have different absolute levels of responding, while the habituation curves shown in **Figure 3B** all sum to 100%.

(3) Use the normalized percentage values to calculate the difference in the percent of responding between the first epoch and the minimum epoch (i.e., the epoch having the lowest percent of responses). This difference is then divided by the amount of time that elapsed between the first epoch and the minimum epoch to produce a rate measure. This is done using the following equation:

$$HR=\frac{{\left(Percent/Epcoh\right)}_{First\,\;Epoch}-{\left(Percent/Epoch\right)}_{Min\,\;Epoch}}{Time_{Between\,\;Epoch}},$$

where HR is the rate of habituation, (Percent/Epoch)_FirstEpoch_ and (Percent/Epoch)_MinEpoch_ are calculated using Eq. 1 for the first epoch and the epoch with the smallest percentage of responses respectively, and Time_BetweenEpochs_ is the amount of time elapsed from the midpoint of the first epoch to the midpoint of the minimum epoch. HRs for the three absolute responding curves shown in **Figure 3A** are depicted in **Figure 3C**. A HR of 1.0 means that while undergoing habituation, for every minute of elapsed session time the response rate will decrease by 1% of the total session response rate. The unit of HR is percent change in response rate per minute (Percent/Epoch/Min).

In practical application, HR can be calculated directly from the data shown in **Figure 3A** using the equation:

$$HR=100\times \frac{Rs_{First\,\;Epoch}-Rs_{Min\,\;Epoch}}{Total_{Rs}\times Time_{Between\,\;Epoch}},$$

where Rs_FirstEpoch_ represents the number of responses made during the first epoch of a session, Rs_MinEpoch_ is the number of responses made during the epoch with the fewest number of responses, TotalRs is the total number of responses made during a session, and Time_BetweenEpochs_ is the amount of time elapsed from the midpoint of the first epoch to the midpoint of the minimum epoch.

## EFFECT OF STIMULANTS ON HRE

**FIGURE 4:**
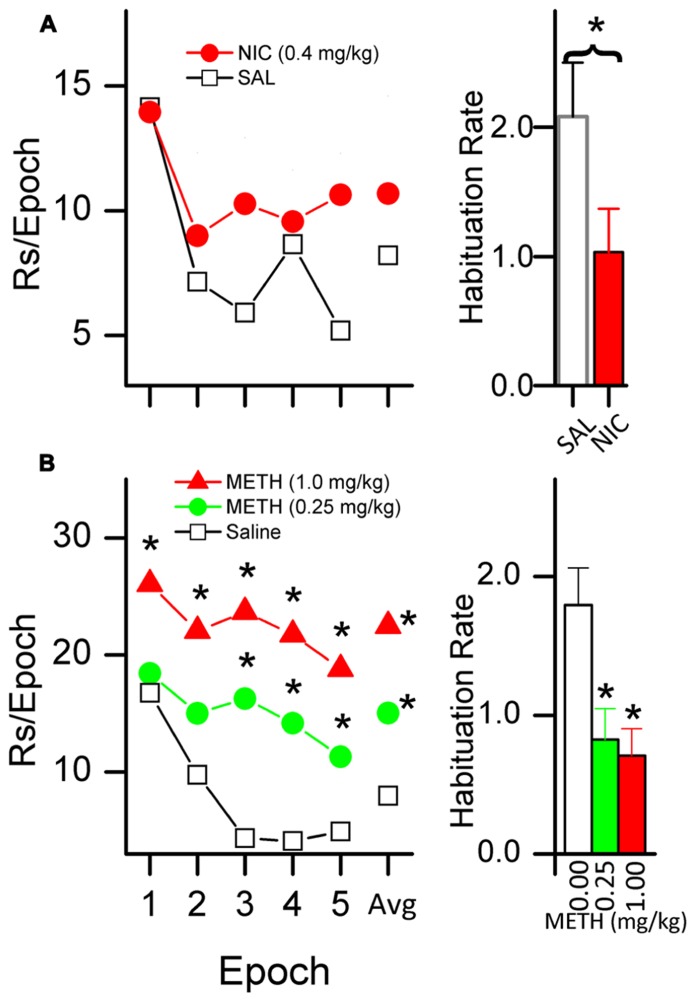
**Nicotine [NIC, (A)] and methamphetamine [METH, (B)] decrease the HRE of sensory reinforcers.** The plots in the left column show responding for a sensory reinforcer (5 s light-onset) after administration of NIC **[(A)**; saline and 0.4 mg/kg] and METH **[(B)**; saline, 0.25, and 1.0 mg/kg]. NIC data shows a 40 min session consisting of five 8-min epochs. METH data shows a 50 min session consisting of five 10-min epochs. The average number of responses per epoch is labeled as data point “Avg.” Both left column plots show that when treated with saline, responding systematically decreased, indicating HRE. Asterisks (*) indicate a within-group difference in responding between saline and drug (*p* < 0.05). Administration of NIC **(A)** and METH **(B)** both slowed HRE. METH increased overall responding but NIC did not. The histogram bars on the right show habituation rate (HR) (see text for explanation of HR).

There is evidence that systemic administration of psychomotor stimulants, including caffeine (Sheppard et al., 2012), d-amphetamine (Glow and Russell, 1973a,b, 1974; Gomer and Jakubczak, 1974; Winterbauer and Balleine, 2007), methamphetamine (Gancarz et al., 2012a; Lloyd et al., 2012b), and nicotine (Chaudhri et al., 2006a; Palmatier et al., 2006; Chaudhri et al., 2007; Raiff and Dallery, 2009), enhance the primary reinforcing effectiveness of sensory stimuli. We have shown for both nicotine (Lloyd et al., in press) and methamphetamine (Gancarz et al., 2012a; Lloyd et al., in press) that the drug-induced increases in responding for sensory reinforcers are accompanied by a decrease in HRE. **Figure 4A** illustrates our findings in rats administered 0.4 mg/kg nicotine, and **Figure 4B** illustrates the effects of 0.25 and 1.0 mg/kg doses of methamphetamine.

Together, the two studies illustrate the central importance of habituation in understanding stimulant effects on reinforcement. Compared to saline, neither drug simply increased the initial degree of reinforcement and then followed the same time course as saline. The higher dose of METH (1.0 mg/kg, **Figure 4B**) was the only drug condition in which the initial reinforcer effectiveness (epoch 1) was greater than that of the placebo. The impact on reinforcer effectiveness for both doses as compared to placebo increased across epochs. In contrast to the steep reduction in response rate in saline-treated rats, active responding is maintained at a relatively stable rate in those receiving 1.0 mg/kg of METH. Thus, it appears that the majority of the drug’s effect at this dose was due to a reduction in HRE.

The situation is even more striking for nicotine and the lower dose of METH (**Figures 4A,B**). Neither of these drug conditions increased initial reinforcing effectiveness above that observed with saline (epoch 1); the entire impact on reinforcer effectiveness for both METH 0.25 mg/kg and for nicotine was due to a slowing of HRE, a reduction in the loss of reinforcer effectiveness over time compared to the saline conditions.

These data indicate that a more detailed behavioral analysis of the within-session pattern of responding may reveal significant drug effects which are obscured by only measuring overall response rate. An important implication of these data is that stimulant drugs may increase the reinforcing effectiveness of sensory stimuli by disrupting or slowing normally occurring HRE. Stimulant-induced decreases in HRE may play a role in the abuse potential of stimulant drugs by sustaining the reinforcing effectiveness of sensory stimuli associated with drug consumption.

### DOES HRE CONTRIBUTE TO STIMULANT ADDICTION?

In support of the hypothesis that HRE plays a role in the drug addiction processes, the ability of stimulant drugs to enhance responding for visual stimuli has been found to be an important factor in rodent drug self-administration studies where the onset (or offset) of a VS is frequently paired with drug delivery. In drug self-administration studies, visual stimuli have often been used to signal drug availability with the assumption that they do not have reinforcing properties of their own. However, at least for nicotine self-administration studies, it has been shown that the reinforcing effects of cue lights interact with the reinforcing effects of drug. In a series of experiments, Donny, Caggiula and coworkers (Caggiula et al., 2001, 2002a,b, 2009; Donny et al., 2003b; Chaudhri et al., 2006a,b,^2007^; Palmatier et al., 2006) have shown that self-administration of nicotine is greatly enhanced by the response-contingent presentation of a VS. Importantly, these studies show that the effects of nicotine on the reinforcing effects of visual stimuli do not depend upon their ability to predict nicotine injections. Rather, they show that increased responding for visual stimuli is due to nicotine-induced increases in the primary reinforcing effectiveness of the visual stimuli. The results of these experiments are extremely important because many investigators do not consider that visual stimuli may have primary reinforcing effects and assume that responding for the visual stimuli that have been paired with drug to be due to conditioned reinforcing effects.

The widespread use of visual stimuli in SA procedures may be an important but largely unexamined aspect of many rodent self-administration studies. We (Gancarz et al., 2011) conducted a limited literature search in the journals (I) Psychopharmacology, (II) Physiology & Behavior, and (III) Pharmacology, Biochemistry and Behavior using the search terms (i) rat, (ii) self-administration, and (iii) cocaine or amphetamine for the years 2007–2010. Of the 101 articles surveyed, 88 (or 87%) used a VS as a cue of drug availability/unavailability in the self-administration procedure (i.e., signaling drug delivery with onset of a house-light, cue light, or both; or flashing or colored lights; or light paired with tones or lever retraction). Surprisingly, only one study recognized use of a VS as a possible confound in interpretation of results (Keiflin et al., 2008).

Therefore, in rodent drug self-administration studies that pair drug delivery with a cue, it is not possible, without proper controls, to determine if subjects are responding to produce the light-onset, administer the drug or some combination. We hypothesize that HRE may play a role in rodent drug self-administration particularly in studies that pair drug delivery with a cue such as light-onset. As has been described above, under normal circumstances the reinforcing effectiveness of sensory reinforcers habituates rapidly. By counteracting the effects of HRE, stimulant drugs maintain the reinforcing effectiveness of visual stimuli and sustain responding. The fact that increases in drug self-administration may be due to increases in the reinforcing effectiveness of the visual cues rather than the reinforcing effectiveness of drug itself should be taken into account.

This same mechanism of disruption (slowing) of normally occurring HRE may contribute to the abuse potential of stimulant drugs such as amphetamines and nicotine in humans. Individuals habituate to sensory and social stimuli to which they are repeatedly exposed, particularly when these events are not associated with unusual or important consequences. If stimulant drugs cancel or counteract the normal process of habituation, stimuli and social interactions to which we are exposed on a daily basis continue to evoke the same excitement and rewarding effects as the first time that we encountered them. Disruption of habituation may result in a more exciting and rewarding subjective experience of the world. Indeed, the effects of abused stimulant drugs are often described as making things more rewarding than they usually are. The HRE concept and data reviewed above suggests that this is not simply an overall increase, but largely a reduction in the natural decrease in reinforcing effectiveness that occurs in the absence of stimulants. It also raises the very interesting possibility that stimulants may be particularly powerful among individuals who are prone to more rapid HRE.

### STIMULANT DRUGS MAY HAVE DIFFERENT EFFECTS ON SENSORY AND CONSUMABLE REINFORCERS.

**FIGURE 5:**
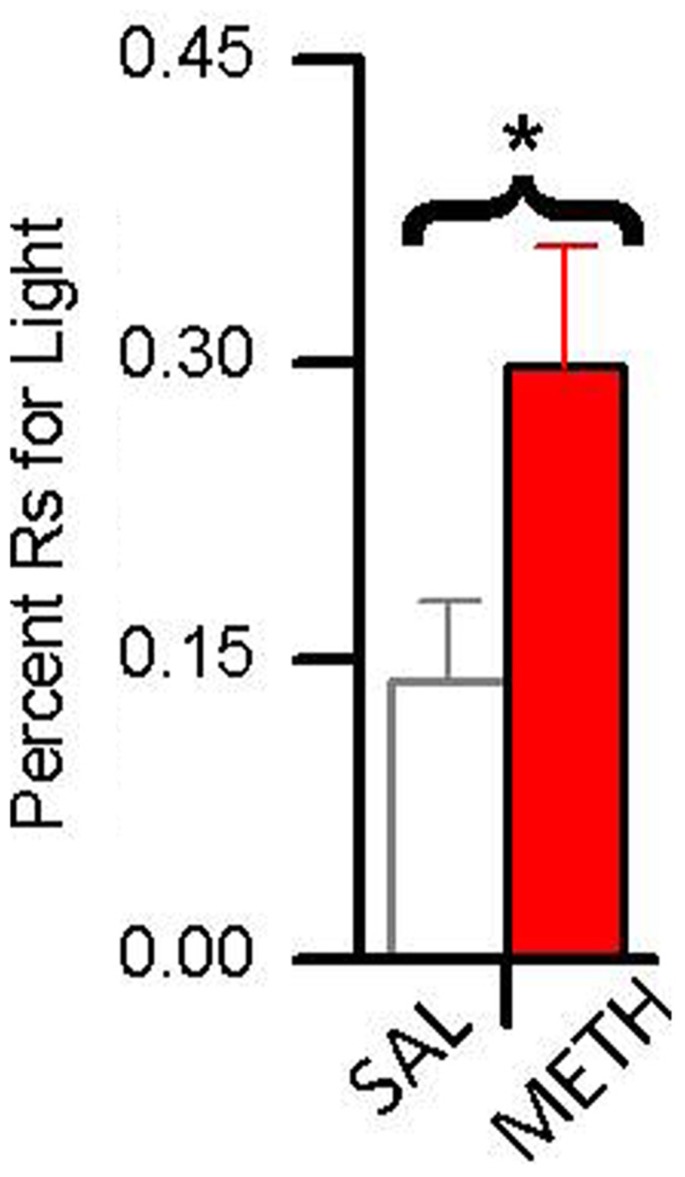
**The effect of 0.5 mg/kg methamphetamine (METH) on choice between a sensory reinforcer and water in water-restricted rats.** The asterisk (*) indicates a difference in responding between saline and METH treated rats (*p* < 0.05) Rats treated with saline responded for the sensory reinforcer <15% of the time. Rats treated with METH responded for the sensory reinforcer about 30% of the time.

In a recent study (Gancarz et al., 2012a), we compared the reinforcing effects of light-onset and water reinforcers using a concurrent VI schedule procedure. Water-restricted rats were pre-exposed to a dark experimental chamber with two snout-poke holes. During a 16 session pre-exposure phase, responding to one hole was reinforced by 0.025 mL water presentations according to a VI 12 min schedule. In the subsequent choice phase, water reinforcement continued unchanged but responding to the alternative hole produced 5 s light-onset presented according to a VI 1 min schedule. During the choice phase, one group of animals was treated with saline and the other group was treated with 0.5 mg/kg METH. As shown in **Figure 5**, while fewer than 15% of responses in the saline group were for the light, about 30% of the METH group responses were for the light.

**FIGURE 6:**
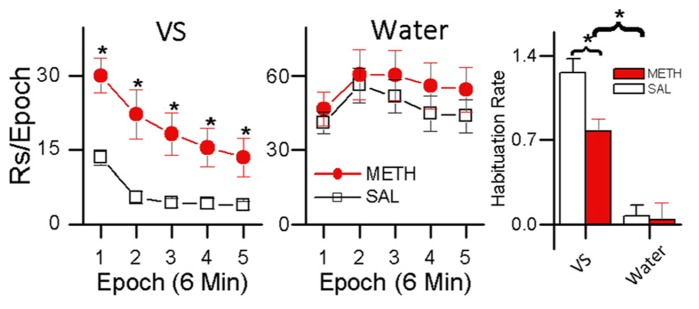
**Within-session analysis of the effect of methamphetamine on concurrent schedule performance for water and sensory reinforcers.** The left plot shows within-session changes in responding for a sensory reinforcer presented according to a VI 1 min schedule in rats treated with METH (0.5 mg/kg) and saline. Asterisks (*) in the left plot indicate differences in responding (*p* < 0.05) The middle plot shows within-session responding for a small (0.025 mL) water reinforcer concurrently presented with the visual stimulus shown in the left plot. The histogram plot on the right shows HRE for sensory and water reinforcers and the effects of saline and METH treatment on HRE. In the histogram, asterisks (*) indicate an overall difference in Habituation Rate between VS and water reinforcers (*p* < 0.05), and a difference in Habituation Rate between the saline and METH treated rats receiving the VS reinforcer (*p* <0.05).

These results indicate two things: (1) evidenced by the rats’ preference for the small less frequent water reinforcer over the more frequent sensory reinforcer, sensory reinforcers have weaker reinforcing effects than biologically important reinforcers; and (2) evidenced by METH-treated rats showing increased preference for light over saline-treated rats, methamphetamine differentially increases the reinforcing effectiveness of sensory reinforcers. A more detailed analysis of these data is presented in **Figure 6**. **Figure 6** shows responding for the sensory and water reinforcers broken down into epochs for saline- and methamphetamine-treated rats. The sensory reinforcer showed clear HRE while the water did not. While METH both increased the rate of responding and decreased the rate of habituation for the sensory reinforcer, it had no significant effect on concurrent responding for the water reinforcer.

A third group was included in the study (data not shown) that received METH injections and water reinforcement but no sensory reinforcement. In this group, responding to the non-water producing snout-poke alternative (inactive) was recorded but had no programed consequences. For this group, METH significantly increased responding for the water alternative without changing response rate to the inactive hole. The control group indicates: (1) that drug-induced adipsia was not a factor; (2) that the response-contingent light was critical for the results; and (3) that rate-dependency does not explain the absence of a significant effect of METH on responding for water (see Gancarz et al., 2012a for details). In summary, this experiment indicates that stimulant drugs such as methamphetamine may have differential effects on sensory and consumable reinforcers and that HRE may be related to this differential effect.

A possible explanation for this effect is that the methamphetamine had a greater effect on the light reinforcer because it had greater HRE. According to this idea very strong reinforcers like water (in water-restricted rats) with small HRE would be less affected by methamphetamine. Reinforcers with small HRE would have less room for stimulants to have a significant effect on HR – but it is possible they would in a longer duration session, with more opportunity for HRE.

Stimulant drugs such as amphetamine are known to induce both anorexia and adipsia in human and non-human animals (Carr and White, 1986). There is evidence that these effects may be mediated by sensory reinforcers that are concurrently available with food or water reinforcers. Carr and White (1986) suggest that stimulant treatment may “increase the tendency for an animal to approach all environmental stimuli as if they were rewarding” and that approaching alternative stimuli competes with the consumption of food and/or water, resulting in decreased consumption (anorexia or adipsia). In support of this hypothesis, they describe an experiment (Carr and White, 1986, p. 21) in which rats pretreated with amphetamine and tested in a stimulus-rich environment consumed less food than amphetamine pretreated rats tested in a barren environment. They interpreted these results as indicating that amphetamine’s anorectic properties were due to potentiation of the rewarding value of stimuli other than food.

Corwin and Schuster (1993) also reported results consistent with this interpretation of amphetamine-induced anorexia. They compared the effects of d-amphetamine on choice between a sensory reinforcer and food in rhesus monkeys. The sensory reinforcer was visual access to the laboratory in which the monkeys were housed. Using a discrete trials procedure, the monkeys chose between food and visual access. They found that lower doses of d-amphetamine increased responding for the sensory reinforcer and decreased choice of food. These results are similar to the results presented in **Figures 5** and **>6**, where METH both increased responding for the VS and decreased HRE for the sensory reinforcer but did not affect responding for water.

To the best of our knowledge, the hypothesis that stimulants cause anorexia by increasing the reinforcing effectiveness of alternative (sensory) reinforcers has not been directly tested in humans, although there is some suggestive data. Leddy et al. (2004) tested the effects of the stimulant methylphenidate on eating pizza in obese men. They found that methylphenidate decreased the amount of pizza that was eaten. Interestingly, methylphenidate did not significantly decrease self-reports of hunger. However, there was no measure of alternative behaviors that may have been increased by methylphenidate. In another study, Perkins et al. (1995) tested abstinent and non-abstinent smokers on a concurrent variable ratio schedule procedure in which they chose between food and money reinforcers. There was an effect of smoking abstinence in a subset of the participants (dietary restrained females). These participants responded less for the food reinforcer and more for the money reinforcer after smoking. However, interpretation of these data in the context of HRE is not possible because within-session changes in responding were not reported, so the effects of smoking on HRE, if any, could not be determined.

### SENSITIZATION

Finally, a possible explanation of the stimulant-induced slowing of HRE is that the drug sensitizes the animal to the effects of the reinforcer. If we consider the rate of responding in the first epoch (before habituation occurs) as an indicator of sensitization, then there is evidence that the rats given 1.0 mg/kg METH (**Figure 4B**) were sensitized to the reinforcing effects of light-onset. The fifth behavioral characteristic of habituation (**Table 1**) states, “The less intense the stimulus, the more rapid and/or more pronounced the behavioral response decrement. Very intense stimuli may yield no observable response decrement.” It follows from this characteristic that a drug-induced increase in the initial reinforcing effectiveness would also induce a slowing of HRE relative to a non-drug control. Thus it is possible that slowing of HRE is a secondary effect of a drug-induced increase in initial reinforcer effectiveness. The data showing the effects of a 1.0 mg/kg dose of METH (**Figure 4B**) on responding for a sensory reinforcer are consistent with this interpretation. This dose of METH produced a large increase in the initial rate (the first epoch in the figure) of responding as well as a slowing of HRE.

In contrast, a sensitization interpretation is not supported by the data for the 0.25 mg/kg dose of METH (**Figure 4B**) because the rate of responding in the first epoch is not different from that of the saline-treated condition. A sensitization interpretation is also not supported by the nicotine data (**Figure 4A**) which also showed a slowing of HRE with no initial increase in reinforcing effectiveness. We conclude that stimulant drugs may have separable effects on sensitization and HRE but that more moderate doses may affect HRE alone.

Future research is needed that examines within-session patterns of responding to firmly establish the relationship between the effects of stimulant drugs on the initial reinforcing effectiveness and HRE. Studies of the effects of stimulant drugs on operant responding most often report only overall average responding for test sessions. These averages are to some degree based on the assumption that the rate of operant responding is constant throughout a test session. The pre-clinical work of McSweeney and her students, and the work we have described above using light-onset as a reinforcer indicate that there are large systematic changes in within-session response rate. HRE provides a systematic approach to characterizing these within-session changes. If our hypothesis that stimulant drugs slow HRE is correct, then it is important to analyze operant responding in a way that allows inspection of within-session changes in responding.

## NEURAL/BEHAVIORAL MODEL OF HRE

**FIGURE 7:**
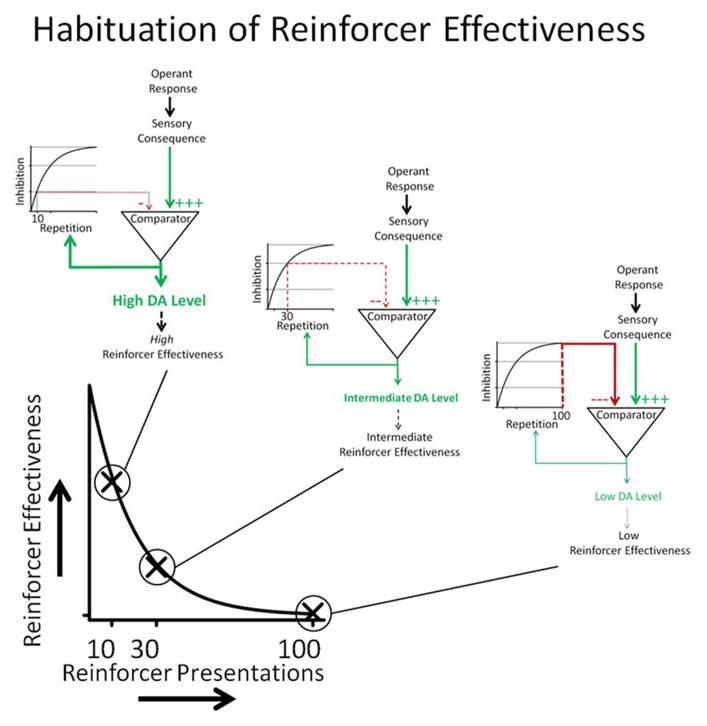
**Operant responses emitted by the organism produce sensory consequences.** If the response-contingent sensory stimulus is unexpected, it increases DA neurotransmission. DA neurotransmission increases the probability that the animal will repeat responses that preceded the onset of the sensory stimulus. Reinforcing effectiveness is operationally defined as response rate. The comparator process depicted in the cartoon determines the novelty of the sensory consequence. Past occurrences of the sensory consequence result in an inhibitory input into the comparator that cancels the effects of the sensory consequence DA neurotransmission. The canceling signal is the integral of the number of response-contingent sensory stimulus presentations so that the strength of the canceling signal is a function of the number of previous sensory reinforcer presentations. The cartoon shows that 10 repetitions caused less intense inhibition than 30 repetitions, which in turn caused less inhibition than 100 repetitions. The conceptual model shows how DA neurotransmission decreases as a function of reinforcer repetition and how changes in DA neurotransmission are hypothesized to underlie HRE.

A conceptual model of habituation of sensory reinforcers is depicted in **Figure 7**. The relationship between DA and sensory consequences depicted in **Figure 7** is largely derived from a model described by Redgrave and Gurney (2006). The Redgrave and Gurney model describes how animals may learn new operant contingencies between responses (actions) and indifferent stimuli. Operant responses emitted by the organism produce indifferent sensory consequences. Although the stimulus itself may not be novel, at least the contingency between response and stimulus consequence is novel (i.e., the sensory stimulus is predicted by the response). The novel sensory contingency increases DA neurotransmission in the basal ganglia which, according to the Redgrave and Gurney model, increases the probability that the animal will repeat the responses that preceded the occurrence of the sensory stimulus. Because reinforcing effectiveness is operationally defined as an increase in response rate, the Redgrave and Gurney model essentially describes a putative neural mechanism that underlies the process of reinforcement. This parsimonious account does not require reference to rewards or incentives.

Novelty and/or surprise have been shown to play an important role in the reinforcing effects of sensory stimuli. Surprise occurs because the occurrence of the stimulus is not predicted by available cues (Kamin, 1969; Blanchard and Honig, 1976; Lloyd et al., 2012b). In the model, repetitive occurrence of response-contingent sensory consequences generates inhibitory signals in a comparator mechanism that counteracts the effects of the sensory stimulus on DA neurotransmission. The strength of the inhibitory signal is an integral of the number of previous sensory reinforcer presentations so that the ability of the inhibitory signal to cancel the effects of the response-contingent sensory stimulus on DA is directly related to how many times it has previously occurred.

The simple comparator model described in **Figure 7** is consistent with memory-based explanations of habituation (Konorski, 1963; Sokolov, 1963; Wagner, 1979). According to memory-based explanations of habituation, perceived stimuli are compared to existing memory. If the perceived sensory stimuli match memory, they are not novel and inhibit the output of the comparator. On the other hand, if the perceived stimuli do not match memory, they are novel or surprising and do not inhibit the output of the comparator. In the model described above, we have avoided the use of a memory construct and simply described the functional relationship between reinforcer repetition and the inhibitory effects of the response-contingent sensory stimulation.

As was mentioned above, the model shown in **Figure 7** is based on a more sophisticated neural model described by Redgrave and coworkers (Redgrave and Gurney, 2006; Redgrave et al., 2008, 2011) that was developed to explain the reinforcing effects of unexpected, indifferent sensory stimuli. Recently Bolado-Gomez and Gurney (2013) have incorporated a quantitative version of this model into a robotic simulation preparation which reproduces the observed behavioral patterns we identified in our published light-reinforcement experiments (Gancarz et al., 2011; Lloyd et al., 2012a). The Redgrave model indicates that sensory consequences (such as light-onset) are detected in sensory areas of the brain (superior colliculus) and cause phasic firing of DA neurons. However, DA neuron activation occurs only if the contingency between the response and indifferent sensory stimulus is unexpected. According to the Redgrave model, increased DA transmission in the basal ganglia causes re-selection of recently emitted responses and thus repetition of the behavior that produced the response-contingent sensory stimulus.

While there is evidence that the neural circuitry described by Redgrave and coworkers underlie increased DA neurotransmission in response to unpredicted response-contingent stimuli, less is known about the neural circuitry that may mediate inhibition of the effects of the sensory reinforcer on DA neurotransmission. The neural structures that cancel out the effects of response-contingent sensory stimuli on DA neurotransmission are largely unknown. Redgrave et al. (2008) have suggested the habenula to be the source of the canceling signal. The lateral habenula may be particularly important because it provides a convergence for neural information from the basal ganglia and limbic forebrain and modulates activation of DA neurons in response to sensory stimuli (Bianco and Wilson, 2009).

The model presented in **Figure 7** greatly over-simplifies the processes underlying HRE; it does, however, serve to highlight two separate neural processes that may underlie changes in the rate of HRE. According to the conceptual model in **Figure 1**, HRE would be *increased* by processes that potentiate inhibition of the effects of previously experienced sensory consequences on DA neurotransmission and by processes that directly *decrease* DA neurotransmission. Conversely, HRE would be decreased by processes that interfere with inhibition of the effects of sensory consequences on DA neurotransmission and by processes that increase DA neurotransmission.

The model described in **Figure 7** is consistent with the declining pattern of responding observed in rats responding to produce light-onset (characteristic 1). Spontaneous recovery (characteristic 2) can also be understood within the general framework of the model if it is assumed that the integrator in **Figure 7** is “leaky.” In this case, inhibitory output from the integrator into the comparator should diminish with time. A leaky integrator would predict spontaneous recovery of responding after periods of no responding. The degree of spontaneous recovery would be a function of how rapidly the integrator “leaked” and the passage of time. Conversely, individuals with very leaky integrators may show very little habituation.

The framework provided by the model outlined in **Figure 7** indicates that drugs that increase DA neurotransmission, such as psychomotor stimulants, should decrease HRE, and that drugs that decrease DA neurotransmission should increase HRE. Presumably, changes in the integrator/comparator function would also lead to changes in downstream DA neurotransmission. A weakness of the model is the lack of specificity with regard to the integrator/comparator function. Research is needed to better understand the neural mechanisms that underlie the integrator/comparator function hypothesized by the model. It seems likely that individual differences in HRE associated with behavioral problems such as those described in the clinical significance section below are due to a dysfunction of the hypothesized integrator/comparator process, and that downstream changes in DA function may be secondary to these effects.

## CLINICAL SIGNIFICANCE OF HRE

Every day, we experience a wide range of sights, sounds, tastes, smells, and tactile stimuli that influence our behavior via operant learning mechanisms. The literature presented above suggests that the reinforcing effectiveness of these sensory reinforcers is subject to habituation. Thus, the rate of HRE may play a critical role in behavioral regulation, and abnormal HRE may result in behavioral dysregulation. Dysfunction of HRE can come in two forms. Abnormally slow HRE would result in excessive or persistent responding to produce sensory consequences and, conversely, abnormally rapid HRE would cause premature cessation of responding for sensory reinforcers. In extreme cases dysfunctional HRE may affect the occurrence and severity of several forms of psychopathology. In this section, we will discuss clinical conditions that may be characterized by slowed and accelerated HRE.

### SLOW HABITUATION

Examples of clinical disorders that may be related to slow-HRE are ASD and obesity. There is good evidence that the stereotypies associated with ASD are operant responses maintained by sensory reinforcement (Lovaas et al., 1987; Rapp and Vollmer, 2005a,b; Cunningham and Schreibman, 2008). From an HRE perspective, the repetitive nature of these operant responses indicates a failure of normally occurring HRE, such that the individual is continually reinforced by sensory properties of the stereotypic behavior (e.g., rocking, head banging, etc.). That said, no study to date has approached ASD-related stereotypies from an HRE framework.

In contrast, there is a large body of work suggesting that obesity is associated with diminished HRE. More than 35% of adults and 15% of youth in the U.S. are obese (Ogden et al., 2012), and obesity is now the second leading cause of preventable death in the U.S. Slow HRE would result in maintenance of food’s reinforcing properties, leading to greater caloric intake. As was described in the introduction, Epstein and coworkers have reported that HRE of food is reduced among obese compared to non-obese people (Temple et al., 2007b; Epstein et al., 2008). In a recent longitudinal study of lean children, those who exhibited slower habituation of operant responding to produce small amounts of food demonstrated greater gains in BMI over the subsequent year (Epstein et al., 2011). These prospective data provide preliminary evidence that individual differences in HRE contribute to the development of obesity, rather than simply covary with obesity.

The HRE framework suggests several ways to impact the reinforcing effectiveness of food. Specifically, the characteristics of habituation (**Table 1**) suggest that interventions that speed HRE of food reduce food intake. For example, HRE theory predicts that varying reinforcers (types of food) should slow HRE, and that repeatedly presenting the same reinforcer should accelerate HRE. Indeed, laboratory experiments demonstrate that increasing dietary variety decreases the rate of habituation on food consumption, while increasing hedonics and salivation, and is associated with increased energy intake (Wisniewski et al., 1992; Temple et al., 2008a). Conversely, Epstein et al. (2011) compared the effects of eating the same, similar, or a variety of food and demonstrated a reduction of energy intake in test groups eating foods with the same or similar characteristics. In a separate study, Epstein et al. (2013) demonstrated long-lasting habituation by assigning the same food more often (daily versus once per week), which resulted in less energy intake over the course of the 5-week study.

These results illustrate how HRE can be applied to develop dietary programs. Several popular diets appear consistent with the principle of reducing dietary variety (e.g., Atkin’s diet, Slim-Fast^®^). In addition, a clinical trial is presently underway to further assess the effects of reinforcer variety in children’s diet. This behavioral intervention is attempting to define the optimal interval for stimulus and variety reduction in diet that will facilitate long-term habituation of calorically dense foods in an effort to address the growing problem of pediatric obesity (ClinicalTrials.gov number NCT01208870).

Another interventional approach to decrease food consumption is to strengthen the reinforcing value of non-food sensory stimuli via stimulant medication. An estimated 15% of Americans take dietary supplements for weight loss (Blanck et al., 2007). Many of these dietary supplements contain stimulant components and are marketed as “boosting energy” and “staving off hunger.” As described in section “Effect of Stimulants on HRE,” stimulants have the potential to induce anorexia and adipsia in humans and animals. Recent work indicates that stimulants may achieve this effect, not by suppressing appetite *per se*, but by increasing the effectiveness of alternative non-consumable reinforcers. In animals, Gancarz et al. (2012a) demonstrated that methamphetamine increases the reinforcing effectiveness of sensory stimuli (light reinforcement) more than concurrently available water reinforcers. We hypothesize that stimulants increase the relative reinforcing value of alternative non-consumable reinforcers which decreases the relative reinforcing effectiveness of food.

Pre-clinical data demonstrating the effects of stimulants on HRE provides insight into the mechanisms of regulation of eating behavior. While stimulant drugs have undesirable side-effects including a potential for abuse, their pre-clinical use allows elucidation of the mechanism by which they decrease overall caloric intake and can suggest behavioral interventions which can be safely implemented. For example, a potential diet may recommend both increasing the variety of alternative (non-food) reinforcers a person experiences (thereby decreasing habituation of non-eating behaviors), while concurrently decreasing the variety of food reinforcers (thereby increasing habituation of eating). The HRE framework predicts that this sort of manipulation would both slow HRE for alternative (non-food) reinforcers and accelerate HRE for food reinforcers.

### RAPID HABITUATION

Clinically, rapid HRE could lead to a lack of responsiveness to typical reinforcers in one’s environment. We hypothesize that rapid HRE is present in ADHD, which is characterized by developmentally inappropriate levels of inattention and/or hyperactivity and impulsivity that creates problems in multiple settings (American Psychiatric Association, 2013). As described below, HRE may inform our understanding of both ADHD psychopathology and the mechanisms of action of the leading evidence-based treatments for the disorder (i.e., behavior therapy and stimulant medication).

Despite the central role of reinforcement in theories of ADHD (Haenlein and Caul, 1987; Sagvolden et al., 2005), there is considerable controversy regarding the exact nature of the dysfunction (Luman et al., 2005; Sonuga-Barke, 2011). From an HRE perspective, poor stimulus control by environmental and social stimuli in children with ADHD may be due to accelerated habituation to reinforcers, rather than a static reinforcement dysfunction, such as an “elevated reward threshold” (Haenlein and Caul, 1987).

The HRE framework developed here provides a relatively novel explanation of ADHD-related deficits. However, this hypothesis has gone largely untested. One exception is the work of Douglas and coworkers (Iaboni et al., 1997) examining the impact of monetary reinforcement on behavior (pressing one of several buttons to turn off a response box light) and heart rate, which tends to accelerate under reinforcement conditions. Douglas et al. found that children with ADHD exhibited faster habituation of heart rate responses to reinforcement than did typically developing children. Although behavioral responding did not show the same pattern, there were only a small number of testing blocks, and each 2-min block was followed by a break, which may have markedly reduced the ability to observe behavioral habituation.

Extensions of this type of work with a simple but boring task, modest reinforcers, and longer testing periods may provide an excellent laboratory analog of real-world conditions under which children with ADHD have great difficulty regulating their behavior (e.g., completing seatwork or homework for 20–60 min). Such a paradigm would also be excellent for testing the degree to which HRE is slowed/accelerated for sensory (and perhaps other) reinforcers in ADHD. Although not presently used to investigate HRE, such a paradigm is already widely used in the ADHD literature. Continuous performance tasks (CPTs) require a response (button press) to infrequent, briefly presented target stimuli over a long period of time (typically 10–30 min), and a decrease in target detection over time is interpreted as a measure of sustained attention, or vigilance. Children with ADHD exhibit a steeper decrease in target detection over time than do typically developing children (e.g., Huang-Pollock et al., 2006; for a review, see Huang-Pollock et al., 2012), which is interpreted as a deficit in sustained attention. However, these results could also be explained by habituation mechanisms. If children with ADHD habituate more rapidly to reinforcers, then the sensory stimuli presented on the computer screen during a CPT would lose their ability to regulate behavior more quickly, leading to a decrement in performance over time.

Mackworth (1969), an early researcher in the study of vigilance tested several predictions based on the characteristics of habituation and concluded in her 1969 text *Vigilance and Habituation* that “the vigilance decrement is a particular example of the process of habituation” (p. 185); she went on to say “These changes probably reflect the reduction in the amount of attention paid to the repetitive stimuli. The changes can be regarded as a reduction in either the quality or quantity of *observing responses* made toward the events of the task” (p. 186). Interestingly, this work has to our knowledge never been applied to the study of ADHD, and the role of HRE in sustained attention receives little consideration in the broader cognitive literature (cf., Ariga and Lleras, 2011; Helton and Russell, 2011).

The HRE hypothesis also addresses the disconnect between neurobiological models and psychological models of reinforcement: “The scarcity of studies testing neurobiological predictions is explained partly by a lack in knowledge of how to test some of these predictions in humans” (Luman et al., 2010). Conversely, psychological models “offer few testable experimental predictions” and are not integrated with neurobiological mechanisms (p. 745). The HRE hypothesis provides a conceptual and empirical bridge between contemporary neural and behavioral models of reinforcement. This is particularly important for understanding the role of reinforcement mechanisms in the leading psychosocial (behavior therapy) and pharmacological (psychostimulant) ADHD treatments (Volkow et al., 2005, 2009; Tripp and Wickens, 2012).

Contingency management approaches, which include the systematic application of reinforcement to enhance the rate of a targeted behavior, are best practice interventions for youth with ADHD. Reinforcement typically involves the contingent presentation of sensory stimuli (e.g., stickers, points, and praise). In these interventions, teachers and parents are typically advised to consistently reinforce the child after every instance of a targeted behavior (i.e., continuous reinforcement; FR1). However, parents often report that reinforcement works initially, but then loses its efficacy over time. As some leading researchers in the field report, “A reinforcer loses its effect when it is given in excessive amounts … ” (Kazdin, 2005) and “Children often become satiated quickly with rewards … resulting in a loss of motivating power as a behavioral-change tool” (Barkley, 1997). Despite this commonly reported clinical concern regarding diminished reinforcing effectiveness over time, these decreases have not been systematically studied. Clinicians often address diminished reinforcing effectiveness by recommending the use of a menu of different reinforcers or introducing new rewards. Paralleling the advances in obesity research noted above, we believe that such treatment programs may be improved through explicit study of the problem of diminishing reinforcer effectiveness within an HRE framework.

The HRE model provides novel avenues for slowing the rate of HRE thereby increasing the rate of a desired behavior. In one of the few studies to have investigated HRE in human participants, Kenzer et al. (2013) were able to increase response rates with a dishabituation manipulation. More studies are needed to investigate how the principles of HRE can be used to improve behavioral interventions for children with ADHD. For example, shorter training sessions interspersed with other activities of recreation (allowing spontaneous recovery) and infusing existing reward menus with theory-based manipulations to capitalize on stimulus specificity (i.e., vary the reinforcers more systematically to proactively prevent habituation, rather than wait for a reinforcer to lose its effectiveness before switching reinforcers) are possible ways to slow habituation in children with ADHD to improve behavior and academic outcomes.

The application of an HRE framework to the understanding of ADHD pathology and treatment is novel, and there are no published studies in this area. The HRE model offers an interpretation of clinical observations as well as clear directions for enhancing treatment. For example, the HRE hypothesis predicts that continuous reinforcement of a behavioral contingency with a single sensory stimulus would be counterproductive because the child would habituate to the reinforcer, and it would lose its effectiveness to control behavior. There is some evidence from pre-clinical studies that the rate of responding for a sensory reinforcer such as light-onset is greater and the rate of habituation is slower when the reinforcer is presented less frequently (see section “Experimental Analysis of HRE with a Light Reinforcer”). These findings are consistent with the principles of HRE discussed in this paper, and provide an explanation for the diminished reinforcing effectiveness over time of contingency management programs used during behavioral intervention for children with ADHD.

In addition to behavioral intervention programs, children with ADHD are often treated with stimulant medication. Experts estimate about 60% of children with ADHD are treated with some type of stimulant medication, and overall about 5% of all children ages 6–18 use prescription stimulant medication (Zuvekas and Vitiello, 2012). Why is stimulant medication used so pervasively and how is it effective in treating children with ADHD? In rodents, methylphenidate blocks the DA transporter and increases DA overflow in the nucleus accumbens and dorsal striatum (Kuczenski and Segal, 1997; Segal and Kuczenski, 1999). Consistent with animal work, Volkow et al. (2002) demonstrated that intravenous stimulant administration in humans increases DA receptor stimulation. Volkow et al. (2004) also demonstrated that oral methylphenidate increased extracellular DA on PET scan in healthy subjects.

An HRE framework provides possible explanations for both ADHD-related impairments and the effectiveness of stimulant medication in the treatment of ADHD. Decreased DA neurotransmission in response to reinforcing stimuli could lead to more rapid HRE causing the child to struggle with maintaining attention and appropriate behavior in school. By blocking DA transporters and preventing DA reuptake, stimulant medication may sustain the effects of reinforcer-induced DA release for longer time intervals, thus slowing HRE. Slowing of HRE would increase stimulus control by social and non-social stimuli, resulting in improved behavior and academic performance. Artificially increasing DA neurotransmission with stimulants slows rapid-HRE in ADHD individuals and maintains responsiveness to reinforcers, which leads to improvements in behavior and academic achievement.

## CONCLUSIONS AND FUTURE DIRECTIONS

We have hypothesized that HRE is a fundamental property of reinforcers, and that reinforcer effectiveness is dynamic. Pre-clinical animal research has almost exclusively focused on the study of powerful consumable reinforcers which exhibit slow-HRE. Pre-clinical researchers have largely used deprivation and other manipulations that mask the effects of habituation. It is important for pre-clinical animal research to go beyond consumable reinforcers. Although there is a large older literature about light reinforcement (for reviews see: Lockard, 1963; Kish, 1966; Berlyne, 1969; Tapp, 1969; Eisenberger, 1972), it is not currently an active area of research. Characterization of the reinforcing effectiveness of a range of repeatedly presented reinforcers, including modest but ubiquitous sensory stimuli, should be an objective of future research. Reinforcers that exhibit rapid-HRE such as light-onset are weak and transient and more difficult to study than strong consumable reinforcers such as food and water. However, it is arguable that weak and transient reinforcers such as light-onset with rapid-HRE make up the majority of reinforcers that regulate day-to-day behavior.

The 10 behavioral characteristics of habituation listed in **Table 1** make testable predictions about operant responding, and research is needed to further test these predictions, particularly in humans. Some of these predictions are surprising. For example, increasing repetition by increasing reinforcer frequency is predicted to decrease response rate. This prediction holds for fast-HRE-reinforcers (see above), but a large body of research with slow-HRE-reinforcers indicates that increasing reinforcing frequency increases response rate. Parametric studies are needed to better understand the dynamic relationship between HRE, reinforcement frequency and response rate.

We have presented evidence that psychomotor stimulant drugs disrupt (slow) normally occurring HRE. This finding has at least two important implications. First, it provides an explanation of the reinforcing effects of stimulant drugs on behavior. The effects of abused stimulant drugs have been described as “making things more rewarding than they usually are.^2^” Stimulant-induced increases in the duration of the reinforcing effects of environmental and social stimuli may underlie this subjective perception. As discussed above, the primary reinforcing effects of cues play an important role in rodent drug self-administration studies, and normally occurring HRE is disrupted by stimulant drugs.

Second, it appears that stimulant drugs may have larger effects on rapid-HRE-reinforcers than slow-HRE-reinforcers. This differential effect predicts a shift in relative preference toward the rapid-HRE-reinforcers. As has been discussed this type of preference shift may underlie the anorectic effects of stimulant drugs. Studies are needed which examine the effect of stimulant drugs on concurrent responding for slow-HRE- (e.g., food or water) and rapid-HRE-reinforcers (e.g., purely sensory stimuli) in both human and non-human subjects to determine the validity of this assertion. For example, in human subjects simple experimental paradigms could measure stimulant effects on within-session decreases in both the reinforcing effectiveness and subjective liking of a reinforcing stimulus (e.g., small amount of food, picture on a computer screen) and include stimulus specificity, dishabituation, and spontaneous recovery tests.

A method for quantitatively measuring the speed of HRE was described. The HR metric is calculated so that absolute differences in response rate do not affect the estimated HR. Future research using this technique may produce estimates of the rate of habituation that are quantitatively comparable across a variety of test situations. Using this measure to index the rate of HRE across a variety of reinforcing stimuli may be particularly important in human studies and clinical settings where a large variety of reinforcers are used.

A conceptual model of HRE was described which has two components, an integrator/comparator component reflecting habituation and a DA neurotransmission component reflecting probability of response repetition. The DA component is well-described by Redgrave and Gurney (2006). In contrast, the neural basis of the integrator/comparator component (where habituation takes place) is unknown. Understanding the neural basis of the integrator/comparator component of the model is important because individual differences in this component may underlie behavioral disorders such as obesity and ADHD which may be caused by abnormal slow-HRE or abnormal rapid-HRE, respectively.

Finally, the HRE concept may provide important insights into the etiology and treatment of behavioral disorders such as obesity and ADHD. Abnormal HRE due to genetic and/or environmental factors may underlie some clinical disorders. For example, recent research indicates that slow-HRE is predictive of obesity, and manipulations that accelerate HRE for food reinforcers may be important treatment components. Conversely, ADHD may reflect accelerated HRE, leading to poor stimulus control, attention, and persistence. The HRE framework suggests specific interventions such as shorter task periods (spontaneous recovery), varying the reinforcer (stimulus specificity), and changing the background context (dishabituation) that can be put to work to slow the loss of reinforcer effectiveness and improve behavioral regulation.

## Conflict of Interest Statement

The authors declare that the research was conducted in the absence of any commercial or financial relationships that could be construed as a potential conflict of interest.

## Footnotes

1 In this manuscript we will use “indifferent” instead of the more commonly used “neutral” to refer to sensory stimuli that do not have any biological importance, because “neutral” implies that these stimuli have no effects, which is clearly untrue when referring to them as reinforcers.

2 When asked to explain why cigarette smoking was addictive on the National Public Radio program Science Friday (September 7, 2012), Dr. Nora Volkow, Director of the National Institute on Drug Abuse, stated, “When you are smoking, everything that is around you is much more salient, much more exciting,” and smoking “makes things more rewarding than they usually are.”
